# Association between fatty acids intake and bone mineral density in adults aged 20–59: NHANES 2011–2018

**DOI:** 10.3389/fnut.2023.1033195

**Published:** 2023-03-17

**Authors:** Ze-Bin Fang, Gao-Xiang Wang, Gui-Zhang Cai, Peng-Xiang Zhang, De-Liang Liu, Shu-Fang Chu, Hui-Lin Li, Hing-Xia Zhao

**Affiliations:** ^1^The Fourth Clinical Medical College of Guangzhou University of Chinese Medicine, Shenzhen, China; ^2^Department of Endocrinology, Shenzhen Traditional Chinese Medicine Hospital, Shenzhen, China; ^3^Shenzhen Traditional Chinese Medicine Hospital Affiliated to Nanjing University of Chinese Medicine, Shenzhen, China

**Keywords:** nutrition, bone mineral density, saturated fatty acids, monounsaturated fatty acids, polyunsaturated fatty acids, NHANES

## Abstract

**Background:**

Previous studies have investigated the link between fatty acid intake and bone mineral density (BMD), but the results are controversial. This study aims to examine the relationship between fatty acid intake and BMD in adults aged 20–59.

**Methods:**

The association between fatty acid consumption and BMD was analyzed using a weighted multiple linear regression model with National Health and Nutrition Examination Survey data from 2011 to 2018. The linearity relationship and saturation value of the connection between fatty acid consumption and BMD were assessed by fitting a smooth curve and a saturation effect analysis model.

**Results:**

The study included 8,942 subjects. We found a significant positive correlation between the consumption of saturated fatty acids, monounsaturated fatty acids (MUFAs), and polyunsaturated fatty acids and BMD. In subgroup analyses that were stratified by gender and race, this association was still shown to be significant. Based on the smooth curve and saturation effect analysis, we found no saturation effect for the three fatty acids and total BMD. However, there was a turning point (20.52 g/d) between MUFAs intake and BMD, and only MUFAs intake >20.52 g/d showed a positive correlation between MUFAs and BMD.

**Conclusion:**

We found that fatty acid intake is beneficial for bone density in adults. Therefore, according to our findings, it is recommended that adults consume moderate amounts of fatty acids to ensure adequate bone mass but not metabolic diseases.

## 1. Introduction

Osteoporosis (OP) is a degenerative disease of the bones that results in weakened bones, weakened microarchitecture, increased fragility, and increased fracture risk (1). The prevalence of OP has been increasing in recent years. According to a study done by the International Society for Clinical Densitometry and the International Foundation for Osteoporosis, more than 70 million Americans will have osteoporosis or bone loss by 2030 (2). With the expansion of human life expectancy and population aging, OP will become a more severe public health problem worldwide (3, 4). Therefore, early detection and intervention of osteoporosis have attracted more and more attention.

As a controllable factor, lifestyle seems to play an important role. Numerous nutrients, especially dietary fatty acid intake, have been shown to potentially effect on bone health (5–10). Studies have shown that the type of fatty acid is critical (8, 10, 11). Saturated fatty acids (SFAs) improve bone health by enhancing osteoclast survival (12), decreasing mesenchymal stem cell differentiation (13), promoting calcium absorption or excretion (14), and suppressing inflammatory gene expression (15). Polyunsaturated fatty acids (PUFAs) affect bone metabolism by combining with PPARγ to induce bone marrow adipocyte differentiation (16), regulate inflammatory response (17), and improve bone marrow microcirculation (18). Additionally, monounsaturated fatty acids (MUFAs) may have possible impacts on prostaglandin activity, influencing bone production and bone resorption (19, 20).

However, after reviewing a lot of literature, we discovered that the effects of PUFAs on bone health have received considerable attention; nevertheless, large-scale clinical investigations are absent. At the same time, we find it interesting that no researcher has focused on the connection between the different types of fatty acids and bone mineral density (BMD). Therefore, we decided to conduct a large-scale cross-sectional study using the National Health and Nutrition Examination Survey (NHANES) Database to investigate the connection between the different types of fatty acids and BMD. This was done to help guide therapeutic efforts.

## 2. Methods

### 2.1. Data source and study population

Data collected by NHANES from 2011 to 2018 was used for this cross-sectional analysis. NHANES adopts an innovative survey mode that combines face-to-face interviews and physical examinations to comprehensively assess the health and nutritional status of residents in the United States. Questions about demographics, socioeconomics, diet, and health were included in the interviews. The medical section addresses medical, dentistry, and physiological examinations as well as laboratory testing performed by qualified medical specialists. In general, these statistics are used to assess the prevalence of diseases and the related risk factors, formulate guidelines for the implementation of practical public health policy, devise health initiatives and services, cultivate fundamental health awareness, and improve the quality of life.

**Figure 1:**
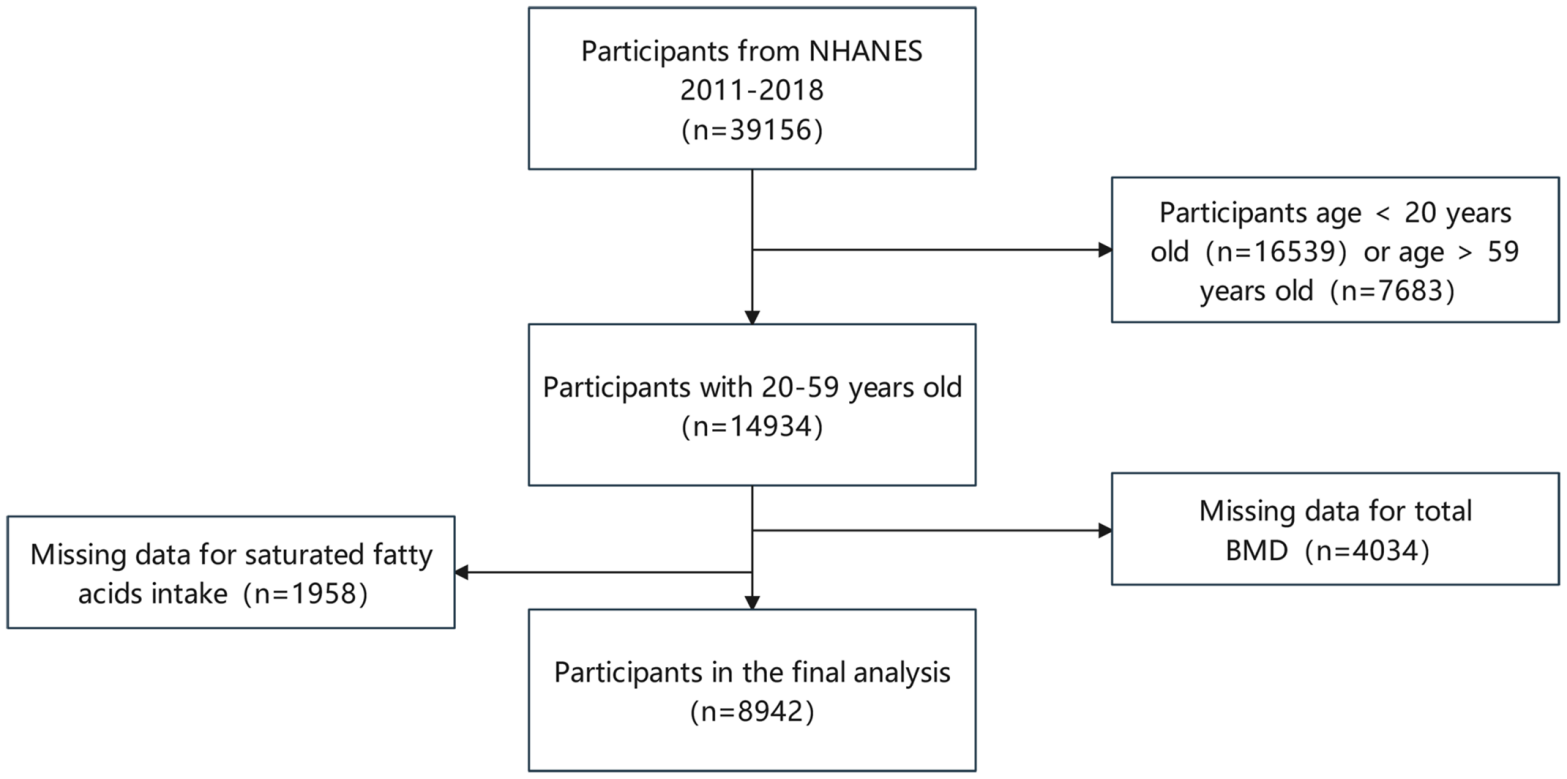
Flowchart of participant selection.

Our analysis comprised 39,156 participants from NHENAS 2011 to 2018, excluding participants under 20 (16,539 individuals) and those beyond 59 (7,683 individuals). Simultaneously, missing data on fatty acids intake (1,958 individuals) and total BMD (4,034 individuals) were excluded. Following the screening mentioned above, we included a total of 8,942 individuals (Figure 1).

### 2.2. Ethics statement

All survey participants were informed of the poll’s specifics and signed an informed consent form. The National Center for Health Statistics Ethics Review Board assessed and authorized the informed consent. Following the completion of official anonymization, all of the data is then made available to the public in order to make the most effective use of these resources. Anyone may access these statistics as long as they adhere to the NHENAS database regulations and are used exclusively for statistical analysis. All studies based on these data should adhere to applicable laws and legislation.

### 2.3. Covariates

The independent variable in our study was daily fatty acids consumption, which was determined through two 24-h food recall interviews. Interviews were conducted in person and over the phone, respectively. In the Mobile Examination Center (MEC), a small room was used to perform the first 24-h recall interviews. Each MEC dietary interview room had a set of uniform measuring parameters. These methods assist respondents in reporting the quantity and variety of food they consume. The information for the second food recall was gathered over the phone and was due 3 to 10 days later. After the participants had finished the in-person interviews, they were provided with measuring glasses, teaspoons, a ruler, and a food model guide to equip them with the tools necessary to record meal portions accurately during the phone interviews. All study participants were required to complete two in-person interviews, each performed by a professional dietary interviewer who spoke Spanish and English fluently. Some categorical variables, such as gender, education level, and moderate exercise, were also included in our study, as well as some continuous variables, including age, body mass index (BMI), the ratio of family income to poverty, alkaline phosphatase, blood calcium, blood phosphorus, blood uric acids, total cholesterol, triglyceride, glycohemoglobin, blood urea nitrogen, serum creatinine, urinary albumin creatinine ratio, total albumin, vitamin D intake, alcohol intake, total SFAs intake, total MUFAs intake, total PUFAs intake, and total BMD. For further details on gathering covariate data and 24-h dietary recall interviews, go to.^1^

### 2.4. Outcome variable

Dual-energy X-ray absorptiometry (DXA) is a clinically recognized method for measuring BMD. Its results can be used for osteoporosis fracture, fracture risk prediction, and drug efficacy evaluation (21). Total BMD, as determined by DXA whole-body scans, served as the outcome variable in our study. Because of their quickness, simplicity, and low radiation exposure are widely used to estimate body composition. In the NHANES, DXA scans of the participants have conducted on a Hologic Discovery Model A densitometer (Hologic, Inc., Bedford, Massachusetts), and data processing was carried out using Hologic APEX (version 4.0) software. Professional technicians who have received training and certificates do the operations above. The official NHANES website has a body composition manual with more information about how the DXA exam works.

### 2.5. Statistical analysis

We used EmpowerStats^2^ and R (3.4.4 version) software for statistical analysis. Typically, we consider a statistical result to be meaningful if the *p* value is lower than 0.05. In this study, all sample sizes are weighted. Continuous variables are reported as Mean ± SD for the comparison of baseline data, and the *p*-value was determined using a weighted linear regression model. The chi-square test was used to figure out the *p*-value, and categorical variables were given as a percentage. Weighted multiple linear regression analyses were performed to assess the effect of intake of the three types of fatty acids on BMD. We established three models of SFAs, MUFAs, and PUFAs intake and BMD, with all confounders (age, gender, race, education level, the ratio of family income to poverty, moderate activity, body mass index, alkaline phosphatase, serum calcium, serum uric acid, total cholesterol, blood urea nitrogen, serum phosphorus, triglyceride, glycohemoglobin, urinary albumin creatinine ratio, and total protein, serum creatinine, vitamin D intake, total saturated fatty acids, total monounsaturated fatty acids, and total polyunsaturated fatty acids) corrected for each model. This was done to improve the reporting of epidemiological observational studies and get the most out of the data. Concurrently, fatty acids consumption was transformed into categorical group data using the quartile approach, and *P* for trend was computed. Segregated according to age, gender, and race, subgroup analysis was performed to assess the association between fatty acids consumption and BMD in varying ages, gender, and race differences. In addition, after controlling for all confounding factors, smooth curve fitting was done, and a saturation effect analysis model was constructed to assess the correlation between the consumption of different fatty acids types and BMD. Results were expressed using turn point, effect–β (95%Cl, *p* value), and the log-likelihood ratio test (LRT test).

## 3. Results

### 3.1. Characteristics of participants

We stratified total bone mineral density by quartiles, Table 1 shows the study sample’s baseline data, including demographic information, physical examination data, laboratory test indicators, and dietary interview information for 8,942 subjects. There are significant differences in age, gender, race, education level, the ratio of family income to poverty, moderate activity, body mass index, alkaline phosphatase, serum calcium, serum uric acid, total cholesterol, blood urea nitrogen, serum creatinine, vitamin D intake, total saturated fatty acids, total monounsaturated fatty acids, and total polyunsaturated fatty acids. However, the difference was not significant in terms of serum phosphorus, triglyceride, glycohemoglobin, urinary albumin creatinine ratio, and total protein.

**Table 1:** Weighted characteristics of the study sample.

Quartiles of total bone mineral density (g/cm^2^)	Lowest quartiles	2nd	3rd	4th	*p-*value
*Age (years)*	42.52 ± 12.55	38.99 ± 11.91	38.72 ± 11.36	39.18 ± 11.29	< 0.001
*Gender (%)*					< 0.001
Male	29.24	41.4	54.65	70.81	
Female	70.76	58.6	45.35	29.19	
*Race/ethnicity (%)*					< 0.001
Mexican American	11.87	10.7	11.85	7.03	
Other Hispanic	8.59	7.55	6.82	5.61	
Non-Hispanic White	62.04	63.35	61.64	57.4	
Non-Hispanic Black	5.64	7.83	10.75	21.65	
Other race - including Multi-race	11.86	10.56	8.95	8.3	
*Educational level, n (%)*					< 0.001
Less than high school	13.64	12.56	12.17	9.32	
High school	22.49	21	24.05	21.04	
More than high school	63.86	66.44	63.78	69.64	
*The ratio of family income to poverty (%)*	2.84 ± 1.63	2.90 ± 1.65	2.90 ± 1.59	3.06 ± 1.63	< 0.001
*Moderate activities (%)*					< 0.001
No	31.67	31.38	29.02	25.26	
Yes	68.33	68.62	70.98	74.74	
Body mass index (kg/m^2^)	27.45 ± 6.56	28.93 ± 6.98	29.14 ± 6.73	30.06 ± 6.50	< 0.001
Alkaline phosphatase (u/L)	71.33 ± 24.16	67.49 ± 22.91	65.52 ± 20.94	64.10 ± 19.62	< 0.001
Serum calcium (mmol/L)	2.35 ± 0.09	2.34 ± 0.08	2.34 ± 0.08	2.35 ± 0.08	< 0.001
Serum phosphorus (mmol/L)	1.22 ± 0.17	1.21 ± 0.18	1.21 ± 0.18	1.20 ± 0.19	0.068
Serum uric acid (umol/L)	294.10 ± 74.32	310.97 ± 75.77	320.33 ± 80.05	340.34 ± 80.47	< 0.001
Total cholesterol (mmol/L)	5.12 ± 1.02	5.00 ± 1.03	4.95 ± 1.03	4.88 ± 1.04	< 0.001
Triglyceride (mmol/L)	1.70 ± 1.20	1.70 ± 1.31	1.74 ± 1.94	1.75 ± 1.71	0.631
Glycohemoglobin (%)	5.52 ± 0.72	5.50 ± 0.84	5.51 ± 0.86	5.56 ± 1.03	0.081
Blood urea nitrogen (mmol/L)	4.43 ± 1.51	4.49 ± 1.44	4.60 ± 1.46	4.77 ± 1.58	< 0.001
Serum creatinine (umol/L)	70.10 ± 25.41	72.86 ± 20.14	77.17 ± 27.79	83.04 ± 37.56	< 0.001
Urinary albumin creatinine ratio (mg/g)	24.88 ± 138.39	18.71 ± 89.00	24.15 ± 226.05	22.54 ± 179.02	0.61
Total protein (g/L)	71.40 ± 4.25	71.51 ± 4.40	71.60 ± 4.36	71.43 ± 4.21	0.385
Vitamin D intake (mcg/d)	8.54 ± 20.59	9.60 ± 22.41	10.98 ± 25.15	12.49 ± 27.36	< 0.001
Alcohol intake (g/d)	4.18 ± 4.19	4.40 ± 4.78	4.46 ± 4.43	4.89 ± 4.60	< 0.001
Total saturated fatty acids intake (g/d)	24.52 ± 11.67	26.20 ± 13.81	27.36 ± 14.05	29.73 ± 14.31	< 0.001
Total monounsaturated fatty acids intake (g/d)	26.44 ± 12.61	28.29 ± 13.98	29.32 ± 14.62	32.44 ± 15.46	< 0.001
Total Polyunsaturated fatty acids intake (g/d)	17.93 ± 9.66	19.11 ± 10.03	19.43 ± 9.97	21.60 ± 11.30	< 0.001

Continuous variables are presented as Mean ± SD, *p*-value was calculated by a weighted linear regression model. Categorical variables are presented as %, *p*-value was calculated by chi-square test.

### 3.2. Relationship between SFAs intake and BMD

**Figure 2:**
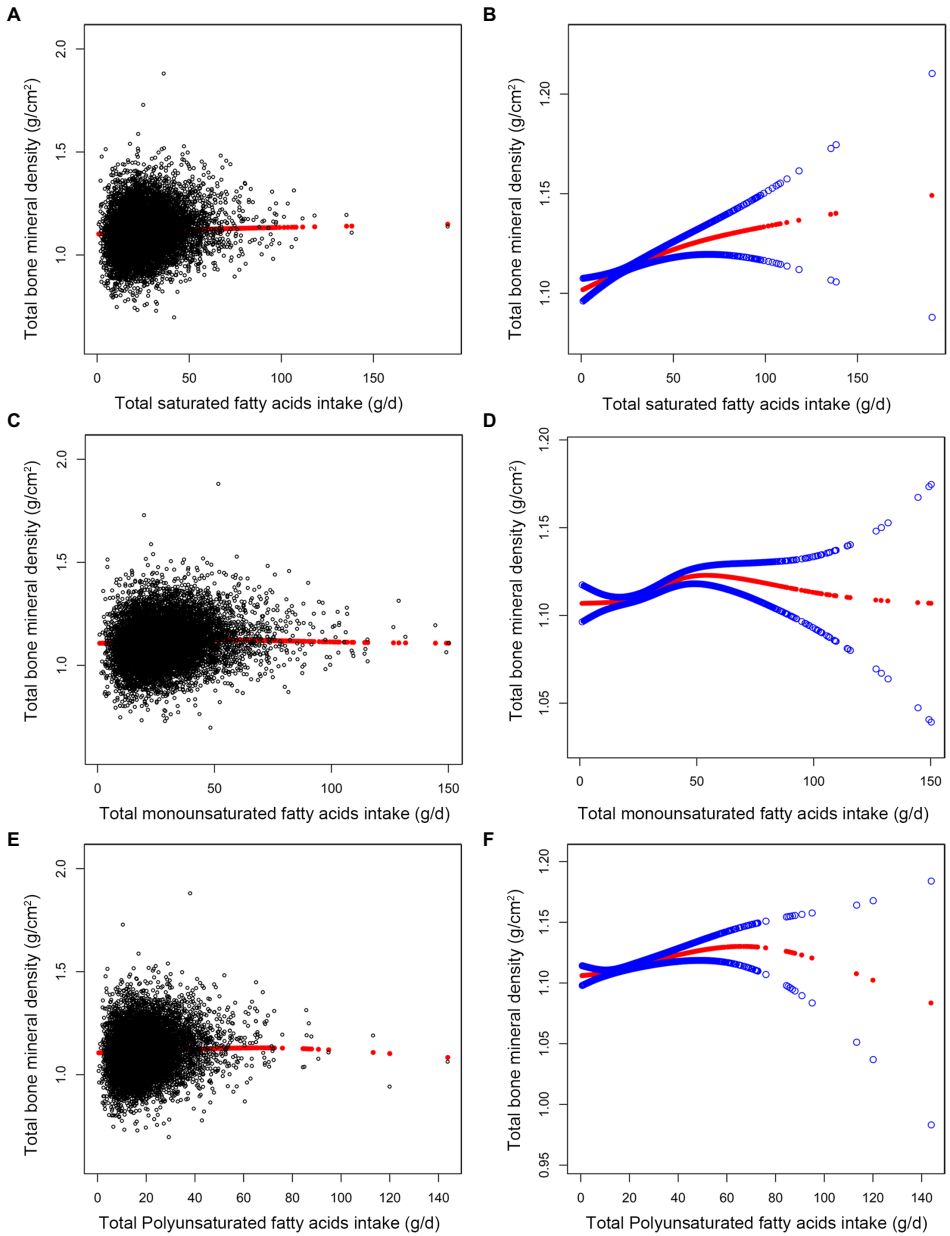
Association between fatty acids intake (g/d) and total bone mineral density (g/cm^2^). **(A,C,E)** Each black point represents a sample. **(B,D,F)** The solid red line represents the smooth curve fit between variables. Blue bands represent the 95% confidence interval from the fit. All confounding factors were adjusted.

Table 2 displays the weighted multiple linear regression model. The result showed that total intake of SFAs was positively linked with total BMD (*p* < 0.001). When the quartile of total SFAs intake was constructed, the lowest quartiles was used as a reference, the trend analysis was statistically significant (*p* for trend = 0.002), and the 4th quartile was significantly positively associated with BMD (*p* < 0.01). After controlling for all confounding variables, the association between total SFAs consumption and BMD was positive and statistically significant in subgroups stratified by age. However, in subgroups stratified by gender, total SFAs intake was statistically positively linked with BMD in male individuals but not in female subjects. In subgroups stratified by race, there was a positive correlation between total BMD and total saturated fatty acids intake among whites, blacks, and other race. These outcomes reach statistical significance. As shown in Figures 2A, B, we found no saturation effect between SFAs and BMD when we performed smooth curve fitting on the revised model.

**Table 2:** Association between fatty acids intake (g/d) and total bone mineral density (g/cm^2^).

Exposure	Total saturated fatty acids intake (g/d) (0.6000–190.0570 g/d)	Total monounsaturated fatty acids intake (g/d) (0.6745–149.8885 g/d)	Total polyunsaturated fatty acids intake (g/d) (0.2825–143.5885 g/d)
	*β*, 95%Cl, *p*-value	*β*, 95%Cl, *p*-value	*β*, 95%Cl, *p*-value
	0.0004 (0.0002, 0.0005)***	0.0003 (0.0002, 0.0005)***	0.0004 (0.0002, 0.0006)***
Quartiles of exposure			
Lowest quartiles	Reference	Reference	Reference
2nd	0.0036 (−0.0023, 0.0094)	−0.0064 (−0.0121, −0.0006)*	0.0034 (−0.0024, 0.0092)
3rd	0.0046 (−0.0014, 0.0105)	−0.0045 (−0.0103, 0.0013)	0.0060 (0.0002, 0.0118)*
4th	0.0101 (0.0039, 0.0163) **	0.0082 (0.0021, 0.0143)**	0.0089 (0.0029, 0.0148)**
*p* for trend	0.002	0.006	0.004
*Stratified by age*			
20–39 years old	0.0003 (0.0000, 0.0005)*	0.0003 (0.0001, 0.0005)**	0.0003 (0.0001, 0.0005)***
40–59 years old	0.0005 (0.0002, 0.0007)***	0.0003 (0.0001, 0.0005)*	0.0003 (0.0001, 0.0005)*
*Stratified by gender*			
Male	0.0004 (0.0002, 0.0006)***	0.0003 (0.0001, 0.0005)**	0.0004 (0.0001, 0.0006)**
Female	0.0002 (−0.0001, 0.0005)	0.0003 (0.0000, 0.0005)*	0.0004 (0.0001, 0.0007)**
*Stratified by race*			
Mexican American	0.0001 (−0.0003, 0.0004)	0.0000 (−0.0004, 0.0004)	0.0001 (−0.0004, 0.0006)
Other Hispanic	0.0000 (−0.0004, 0.0005)	−0.0000 (−0.0005, 0.0004)	0.0003 (−0.0003, 0.0009)
Non-Hispanic White	0.0003 (0.0000, 0.0006)*	0.0003 (0.0000, 0.0005)*	0.0003 (−0.0001, 0.0006)
Non-Hispanic Black	0.0006 (0.0003, 0.0010)**	0.0005 (0.0002, 0.0009)**	0.0005 (0.0000, 0.0010) *
Other race	0.0009 (0.0005, 0.0013)***	0.0006 (0.0003, 0.0010) ***	0.0013 (0.0008, 0.0017) ***

All confounding factors (age, gender, race, education level, the ratio of family income to poverty, moderate activity, body mass index, alkaline phosphatase, serum calcium, serum uric acid, total cholesterol, blood urea nitrogen, serum phosphorus, triglyceride, glycohemoglobin, urinary albumin creatinine ratio, total protein, serum creatinine, vitamin D intake, alcohol intake, total saturated fatty acids, total monounsaturated fatty acids, and total polyunsaturated fatty acids) were adjusted.

The model is not adjusted for the stratification variable itself in the subgroup analysis.

*p < 0.05, **p < 0.01, ***p < 0.001.

### 3.3. Relationship between MUFAs intake and BMD

As shown in Table 2, we found a significant positive connection between total MUFAs intake and BMD (*p* < 0.001) using the revised model. When quartiles of total MUFAs were quantified, the 2nd quartile was negatively connected with BMD (*p* < 0.05). In contrast, the 4th quartile was positively correlated with BMD (*p* < 0.01), and the trend analysis was statistically significant (*p* for trend = 0.006). There was a statistically significant correlation between total MUFAs intake and BMD across age groups in subgroups stratified by age and gender. BMD was linked to total MUFA intake in whites, blacks, and people of other race, these associations are statistically significant.

We found a turning point between total MUFAs intake and BMD using a smooth curve fit inside a model that controlled all covariates Figures 2C, D. According to the saturation effect analysis model, the effect value for total MUFAs consumption was 20.52 g/d (Table 3). Taken together, the connection between MUFAs intake and total BMD showed an inverted U-shaped curve.

**Table 3:** Saturation effect analysis of fatty acids intake (g/d) and total bone mineral density (g/cm^2^).

	Total bone mineral density (g/cm^2^)
*Turn point of total saturated fatty acids intake (g/d)*	*33.67*
< 33.67, effect 1	0.000 (−0.000, 0.000) 0.2454
> 33.67, effect 2	0.001 (0.000, 0.001) 0.0002
The effect difference	0.000 (−0.000, 0.001) 0.1060
Predicted value of the equation at the folding point	1.123 (1.119, 1.127)
Log likelihood ratio test	0.105
*Turn point of total monounsaturated fatty acids intake (g/d)*	*20.52*
< 20.52, effect 1	−0.001 (−0.001, 0.000) 0.0546
>20.52, effect 2	0.000 (0.000, 0.001) <0.0001
The effect difference	0.001 (0.000, 0.002) 0.0036
Predicted value of the equation at the folding point	1.101 (1.098, 1.105)
Log likelihood ratio test	0.004
*Turn point of total polyunsaturated fatty acids intake (g/d)*	*6.23*
<6.23, effect 1	−0.003 (−0.008, 0.002) 0.2212
>6.23, effect 2	0.000 (0.000, 0.001) <0.0001
The effect difference	0.003 (−0.001, 0.008) 0.1644
Predicted value of the equation at the folding point	1.093 (1.090, 1.097)
Log likelihood ratio test	0.164

All confounding factors were adjusted.

### 3.4. Relationship between PUFAs intake and BMD

In the fully adjusted model, total PUFAs intake was also found to be positively associated with BMD (*p* < 0.001). When total PUFAs intake was analyzed by quartile, fatty acids intake was positively associated with total BMD in the 3rd group (*p* < 0.01) and the 4th group (*p* < 0.01), and the trend analysis was statistically significant (*p* for trend = 0.004) (Table 2). In subgroups stratified by age and gender, the positive association between total PUFAs intake and total BMD remained statistically significant. In subgroups stratified by race, we observed this positive association only in blacks and other genders. These outcomes possess statistical significance. As shown in Figures 2E, F, we found no saturation effect between PUFAs and BMD when we performed smooth curve fitting on the revised model.

## 4. Discussion

We analyzed the association of fatty acid intake with BMD using data on adults aged 20–59 years in the NHANES from 2011 to 2018. Three classes of fatty acids (SFAs, PUFAs, and MUFAs) were favorably linked with BMD in this cross-sectional study of 8,942 people. In this study, we analyzed fatty acid intake by quartile and found that higher fatty acid intake was associated with better bone health within a certain range of fatty acid intake. Furthermore, saturation effect model analysis and smooth curve fitting showed that total MUFAs had an inverted U-shaped relationship with BMD, with a turning point of 20.52 g/d, while other fatty acids had a linear relationship with BMD once confounding factors were taken into account. When total MUFAs were higher than 20.52 g/d, there was a beneficial association between MUFAs intake and BMD (*p* < 0.0001). Study (22) has shown that MUFAs activate peroxisome proliferators receptor-β/δ, regulating the RANKL signaling pathway, and inhibiting osteoclast formation.

Currently, PUFAs have received the most attention in bone health investigations, but the results are controversial. In the ORENTRA experiment, Jørgensen et al. (23) supplied omega-3 fatty acids to kidney transplant recipients and olive oil to the control group. After 44 weeks of treatment, omega-3 fatty acids had no significant effect on BMD. The researchers believe that it may be due to the threshold effect of n-3 PUFAs on BMD. In a retrospective study of 275 healthy women from Japan, Kuroda et al. (24). used multiple linear regression analysis to show that omega-3 fatty acid intake contributes to hip BMD. Results from this study showed that the connection between PUFAs intake and BMD was stronger in the 3rd and 4th quartiles of the distribution, but not in the 2nd quartile. We believe that PUFAs intake is positively correlated with BMD only when PUFAs intake reaches a certain threshold, which seems to be consistent with previous research. Notably, a 5-year longitudinal study (25) found that a higher intake of PUFAs and MUFAs was linked to lower BMD in the femoral neck, even after controlling for possible confounding factors, these associations are statistically significant. This contradictory conclusion may be owing to the limited sample size of the population included in this study. Similarly, in animal study (26), BMD was significantly reduced in rats fed an atherogenic diet, and monounsaturated fatty acids ameliorated these changes but remained lower than in controls. Little research has been conducted on the correlation between saturated fatty acid consumption and BMD to date, and there has been a dearth of large-scale investigations into the topic. Because of this, we conducted this extensive retrospective study and found that consuming saturated fatty acids actually improves BMD.

In addition to the regulation of bone metabolism, fatty acids have other biological effects, such as omega-3 PUFAs to protect cardiovascular (27) and nerve (28), anti-tumor (29), possibly by inhibiting inflammation, reducing oxidative stress, regulating cell apoptosis and other mechanisms. A meta-analysis with 23 literature showed that when SFAs intake >17 g/d, there will be a clear protective effect of type 2 diabetes (30). Data from the literature indicates that diabetes is a high risk factor for osteoporosis (31). According to a number of studies, those who suffer from diabetes have lower bone mineral density than those who do not suffer from the condition (32, 33). In addition, the ratio of PUFAs (e.g., omega-6, omega-3) also affects metabolism. Studies have shown that a high intake of omega-6 fatty acids or a high omega-6/omega-3 nutrient ratio is linked to an increased risk of obesity. Obesity is also a protective factor for bone density, which partially explain why PUFAs are good for BMD (34, 35). In addition, BMD has been shown to be higher in those with adequate fat intake than in those with insufficient fat intake (36).

We performed weighted multiple linear regression analysis and smooth curve fitting analysis with data from 8,942 participants and found that fatty acid intake in adults aged 20–59 was beneficial to bone mineral density, which is also associated with osteoporosis. Prevention provides dietary guidance. However, our study also has some flaws. Because this is a cross-sectional study, it cannot show that the association between fatty acid consumption and BMD is caused by one or the other. More prospective clinical studies and basic research are needed to back up these results. According to our findings, the positive correlation between total BMD and MUFAs intake occurs only when the MUFAs intake is >20.52 g/d. We have studied a sizable amount of literature. However, to our knowledge, the saturation effect and threshold between MUFAs and BMD are not supported by any pertinent data. The exact mechanism is yet unknown, and more studies are needed to confirm it. There is no literature on the saturation effect between SFAs, PUFAs, and BMD. Therefore, in the future, we suggest carrying out a larger prospective study on SFAs, PUFAs, and BMD to further understand the causal relationship between fatty acids and BMD. Since there are only total SFAs, PUFAs, and MUFAs intakes in the NHANES database, but no specific fatty acid intakes, such as the specific intakes of n-3 and n-6 PUFAs, we suggest that future studies should focus on the association between specific fatty acids and BMD.

In conclusion, SFAs, MUFAs, and PUFAs intake were positively associated with BMD, and the associations persisted in subgroups stratified by age, gender, and race in this study. Notably, when fatty acid intake was quartiled, MUFAs in the 2nd quartile were negatively correlated with BMD and those in the 4th quartile were positively correlated. Meanwhile, this research found that MUFAs were positively correlated with BMD, but there was a threshold. Therefore, according to our findings, it is recommended that adults consume moderate amounts of fatty acids to ensure adequate bone mass but not metabolic diseases.

## Data availability statement

Publicly available datasets were analyzed in this study. This data can be found here: https://www.cdc.gov/nchs/nhanes/.

## Ethics statement

All survey participants were informed of the poll’s specifics and signed an informed consent form. The National Center for Health Statistics Ethics Review Board assessed and authorized the informed consent. Following the completion of official anonymization, all of the data is then made available to the public in order to make the most effective use of these resources. Anyone may access these statistics as long as they adhere to the NHENAS database regulations and are used exclusively for statistical analysis. All studies based on these data should adhere to applicable laws and legislation. Written informed consent for participation was not required for this study in accordance with the national legislation and the institutional requirements.

## Author contributions

Z-BF and G-XW contributed equally to this study and made contributions to data collection, curation, statistical analysis, and manuscript writing and revision. G-ZC and P-XZ contributed to the statistical analysis. D-LL, S-FC, and H-XZ supervised the study and contributed to the polishing and reviewing of the manuscript. S-FC provided financial assistance for this research. H-LL supervised, wrote the review, and edited this study. All authors contributed to the article and approved the submitted version.

## Funding

H-LL was supported by the Shenzhen Municipal Science and Technology Innovation Council (JCYJ20170817094838619). S-FC was supported by the Natural Science Foundation of Guangdong Provincial (No. 2019A1515110108), National Natural Science Foundation of China (grant number. 82104759), and the Shenzhen Municipal Science and Technology Innovation Council (No. JCYJ20180302173821841). The funder had no role in study design, data collection and analysis, decision to publish, or preparation of the manuscript.

## Conflict of interest

The authors declare that the research was conducted in the absence of any commercial or financial relationships that could be construed as a potential conflict of interest.

## Publisher’s note

All claims expressed in this article are solely those of the authors and do not necessarily represent those of their affiliated organizations, or those of the publisher, the editors and the reviewers. Any product that may be evaluated in this article, or claim that may be made by its manufacturer, is not guaranteed or endorsed by the publisher.

## Footnotes

^1^ www.cdc.gov/nchs/nhanes/

^2^ http://www.empowerstats.com
